# Acupuncture regulates neuroinflammation: microglial glucose metabolism reprogramming as a potential mediating mechanism

**DOI:** 10.3389/fimmu.2026.1853257

**Published:** 2026-07-01

**Authors:** Ruqi Zhang, Xueke Gao, Han Wang, Shengchun Wang

**Affiliations:** 1 College of Acupuncture, Moxibustion and Tuina, Shandong University of Traditional Chinese Medicine, Jinan, China; 2 College of Physical Education and Health, Henan Mechanical and Electrical Vocational College, Zhengzhou, China; 3 Department of Acupuncture, Physiotherapy and Rehabilitation, Shandong Provincial Hospital Affiliated to Shandong First Medical University, Jinan, China

**Keywords:** acupuncture, glucose metabolism reprogramming, glycolysis, microglia, neuroinflammation

## Abstract

Neuroinflammation is a common pathological feature shared by various central nervous system disorders. As the intrinsic immune cells of the brain, microglia exhibit activation states and functional phenotypes that are regulated by glucose metabolism reprogramming. Under physiological conditions, microglia primarily rely on oxidative phosphorylation to maintain energy homeostasis and immune surveillance. Upon pathological stimulation, however, microglia undergo metabolic reprogramming toward a pro-inflammatory mode dominated by glycolysis. This shift establishes a positive feedback loop between glycolysis and inflammation, which exacerbates neuronal damage. Acupuncture, as a classic non-pharmacological intervention, can suppress neuroinflammation by modulating microglial glucose metabolism reprogramming through multiple pathways. Recent studies have revealed that acupuncture can regulate glucose transporter expression and restore the activity and nuclear translocation of rate limiting enzymes such as hexokinase 2 and pyruvate kinase M2. It also modulates signaling axes including AMP activated protein kinase, mammalian target of rapamycin, and hypoxia inducible factor 1 alpha. These effects contribute to the phenotypic transition of microglia from a glycolysis dependent pro-inflammatory state to an oxidative phosphorylation dominated anti-inflammatory state. In addition, acupuncture exerts indirect anti-inflammatory effects by improving systemic glucose metabolism, optimizing cerebral blood flow, and regulating neurotransmitter balance, thereby reshaping the energy substrate composition of the brain microenvironment. This review systematically summarizes the important role of microglial glucose metabolism reprogramming in neuroinflammation and explores the potential molecular mechanisms through which acupuncture modulates this process. From a metabolic immune perspective, this work provides a new theoretical basis for acupuncture mediated alleviation of neuroinflammation.

## 1 Introduction

Neuroinflammation is a complex immune response of the central nervous system (CNS) to various injuries or pathological stimuli (1). Under physiological conditions, moderate inflammation helps clear pathogens and damaged cells, thereby promoting tissue repair (2). However, when inflammation persists or becomes overactivated, it evolves into chronic neuroinflammation, which subsequently drives neuronal damage and dysfunction (3). In recent years, a growing body of evidence has identified neuroinflammation as a key factor promoting the onset and progression of many diseases (4). Neuroinflammation does not arise from the isolated action of a single cell type. Instead, it is a complex network reaction co driven by dynamic interactions between intrinsic components of the central nervous system, such as microglia, astrocytes, neurons, oligodendrocytes, endothelial cells, and the blood brain barrier, as well as peripherally infiltrating immune cells. In this process, microglia, as the intrinsic immune cells of the CNS, serve as an important mediator of neuroinflammation. They are rapidly activated upon sensing danger signals and subsequently secrete inflammatory cytokines. In relevant animal models, microglia are markedly activated (5). Meanwhile, the expression levels of pro-inflammatory factors such as tumor necrosis factor alpha (TNF-α) and interleukin 1 beta (IL-1β) are also significantly elevated (6). This persistent chronic neuroinflammatory environment not only directly exacerbates neuronal damage in the brain but also interacts with pathological changes such as alpha synuclein aggregation, thereby accelerating disease progression (7).

The functional state of microglia influences changes in neuroinflammation (8). The activation phenotype of microglia is closely associated with their internal metabolic patterns (9). Long term metabolic dysregulation can lead to chronic inflammation and impair the repair function of microglia, which represents an important pathological mechanism in several diseases (10). Thus, glucose metabolism reprogramming in microglia is not merely a concomitant phenomenon following their activation but rather an upstream mechanism that drives the initiation and persistence of neuroinflammation. Therefore, targeting this process may represent an emerging research area for disease treatment (11).

Acupuncture therapy, as an important component of traditional medicine, has garnered widespread attention and high recognition worldwide due to its unique theoretical framework and proven clinical efficacy (12, 13). The mechanisms underlying its therapeutic effects, particularly the specific pathways and mechanisms through which acupuncture acts on neurological disorders, have long been a focal point of medical research (14, 15). Modern studies have confirmed that acupuncture exerts neuroprotective effects through multiple pathways, especially in suppressing neuroinflammation (16). However, whether acupuncture can exert anti inflammatory effects by intervening in microglial metabolic reprogramming remains systematically unexplored. Therefore, this review aims to synthesize the research progress on microglial metabolic reprogramming in neuroinflammation and to explore the potential scientific basis for acupuncture mediated regulation of this process. From a metabolic immune perspective, this work provides a new theoretical foundation and potential targets for acupuncture based treatment of diseases.

## 2 Microglia physiological function and metabolic characteristics

Microglia originate from myeloid precursor cells in the embryonic yolk sac. They colonize the brain parenchyma during early development and maintain population stability in the adult brain through self renewal (17). Under healthy physiological conditions, these cells continuously extend and retract their processes. This allows them to perform uninterrupted immune surveillance of the brain parenchyma and rapidly detect microenvironmental disturbances such as pathogens, damaged cells, or abnormal protein aggregates (18). Functionally, microglia can sensitively recognize pathogen associated molecular patterns (PAMPs) and damage associated molecular patterns (DAMPs) through pattern recognition receptors such as Toll like receptors, thereby rapidly initiating innate immune responses at the early stage of infection or injury (8). Concurrently, microglia participate in synaptic pruning during neurodevelopment and plasticity, eliminating excess and weakened synapses via phagocytic activity to shape neural architecture and maintain normal neurological function (19). Furthermore, as efficient scavenger cells, microglia promptly clear metabolic waste, apoptotic debris, and misfolded proteins through phagocytosis, thereby preserving homeostasis within the brain microenvironment (8).

The realization of these complex functions depends on an efficient and flexible energy metabolism network. However, the central nervous system possesses unique metabolic barriers, as energy substrates such as glucose, fatty acids, and amino acids cannot freely cross the blood brain barrier, resulting in spatiotemporal heterogeneity of nutrient supply within the brain parenchyma. To address this challenge, microglia have evolved a high degree of metabolic flexibility. This flexibility allows them to dynamically adjust their energy metabolism pathways based on changes in environmental substrates (20). Studies have shown that resting microglia simultaneously express key genes from multiple energy pathways, including glycolysis, oxidative phosphorylation, fatty acid oxidation, and amino acid metabolism (21). Microglia not only express glucose transporters (GLUT) (22), but also monocarboxylate transporters (MCT) for lactate and ketone body uptake (23), as well as sodium coupled neutral amino acid transporter 1 for amino acid uptake (24). Under physiological conditions with stable energy demands, microglia mainly rely on the tricarboxylic acid cycle and oxidative phosphorylation to generate ATP. This mode of energy production is efficient and sustainable. It meets the energy requirements for maintaining complex branched morphology and performing basic immune surveillance (25, 26). This process utilizes glucose and fatty acids as substrates to produce ATP (27). However, when glucose supply in the microenvironment is restricted, microglia can rapidly initiate alternative energy utilization pathways and adjust their metabolic profiles to meet different functional demands. Studies have shown that glutamine serves as an important alternative substrate for maintaining microglial metabolic homeostasis. Even under glucose free culture conditions, glutamine can be converted into α-ketoglutarate to enter the tricarboxylic acid cycle, thereby sustaining mitochondrial respiration, cell viability, and phagocytic function (20). Furthermore, microglia can uptake and utilize lactate, oxidizing it to pyruvate via lactate dehydrogenase B to fuel the tricarboxylic acid cycle, thereby supporting functions such as cell proliferation, migration, and phagocytosis (28).

Conversely, when microglia encounter pro-inflammatory stimuli such as PAMPs, they undergo a characteristic metabolic reprogramming. In this state, they preferentially adopt aerobic glycolysis as their primary energy source (29). Even under oxygen rich conditions, microglia still shift their metabolism in response to inflammatory stimulation. This form of metabolic reprogramming rapidly supplies both ATP and biosynthetic precursors required for producing pro-inflammatory cytokines, thereby supporting cytoskeletal rearrangement, proliferation, and production of inflammatory mediators (10). In disease states, microglial metabolic homeostasis is disrupted, and their metabolic reprogramming exhibits pathological features, becoming a key driver of neuroinflammation and disease progression.

## 3 Reprogramming of glucose metabolism in microglia

**Figure 1:**
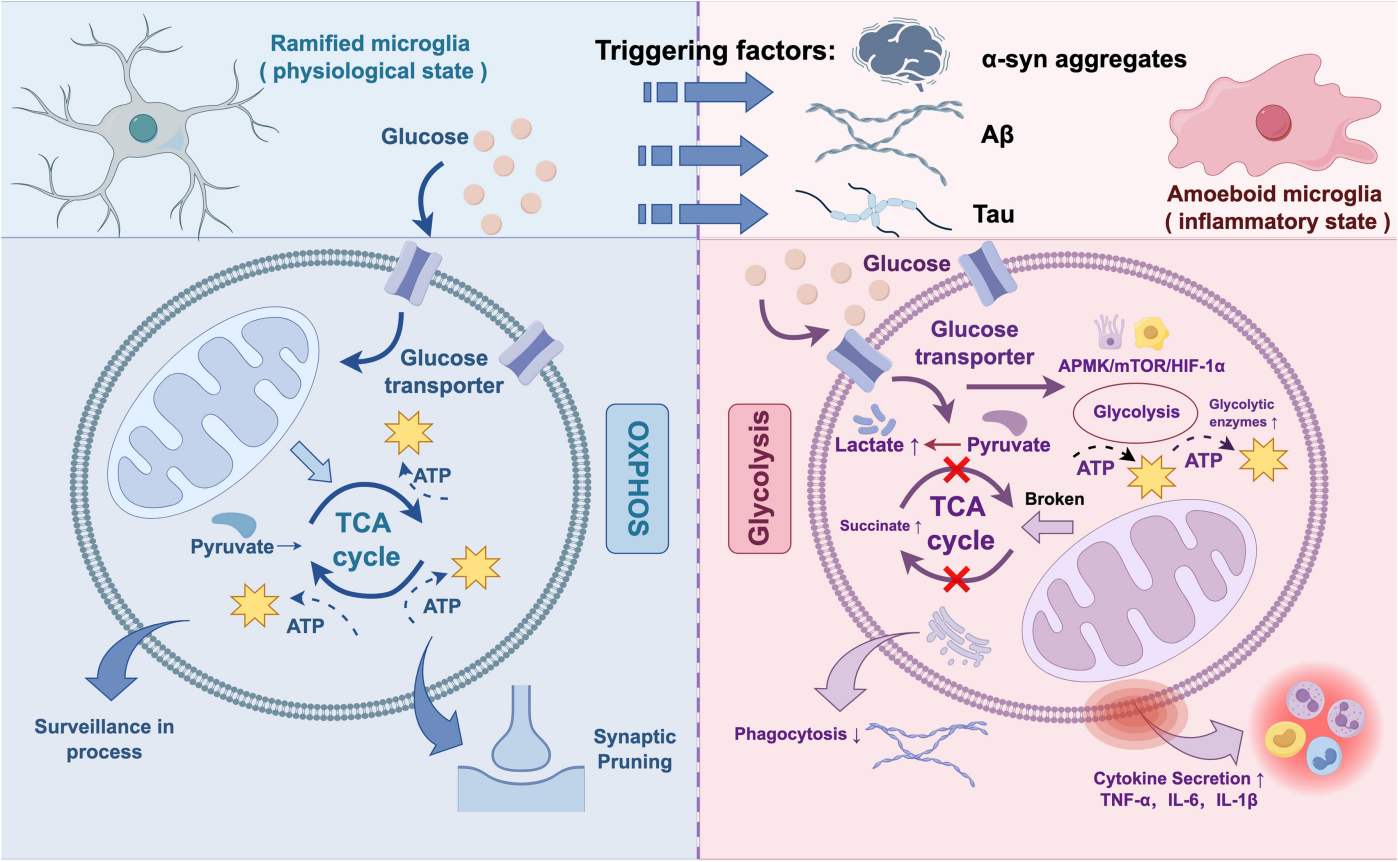
Glycolytic reprogramming of microglia under physiological and inflammatory conditions. Under physiological conditions, ramified microglia take up glucose via glucose transporters and generate ATP through oxidative phosphorylation coupled with the tricarboxylic acid (TCA) cycle, thereby maintaining functions such as immune surveillance of the central nervous system microenvironment and synaptic pruning. When exposed to triggers including alpha synuclein aggregates, amyloid beta (Aβ), and Tau protein, microglia undergo metabolic reprogramming. This process is characterized by persistently increased glucose uptake, enhanced glycolytic flux, elevated lactate production, and upregulation of glycolytic enzyme activities. Simultaneously, accumulation of the TCA cycle intermediate succinate occurs, accompanied by abundant ATP generation. This metabolic shift is associated with signaling pathways such as AMPK/mTOR/HIF-1α. Ultimately, these changes lead to markedly increased secretion of proinflammatory cytokines including tumor necrosis factor alpha (TNF-α), interleukin 6 (IL-6), and IL-1β, thereby exacerbating neuroinflammation. In addition, microglial functions such as phagocytosis, immune surveillance, and chemotaxis are reduced. The figure was made using www.figdraw.com, and the authorization code is RROAP8804c.

Metabolic reprogramming is a process by which cells systematically adjust their energy pathways to adapt to microenvironmental changes or meet specific functional demands. Microglia can shift between anti inflammatory (M2 type) and pro inflammatory (M1 type) phenotypes in response to alterations in the brain microenvironment. With the advancement of single cell transcriptomics, studies have revealed that microglia exhibit a continuous functional spectrum rather than discrete binary end states during neuroinflammation. Multiple specific subpopulations beyond the traditional M1/M2 framework have been identified. Nevertheless, a transition between anti inflammatory and pro inflammatory states still exists. This metabolic transition resembles the Warburg effect described in classical immunometabolism and contributes to the initiation and progression of various neurodegenerative diseases (30). The molecular mechanism of microglial glucose metabolic reprogramming is shown in **Figure 1**. However, metabolic reprogramming in microglia is not limited to glucose metabolism. It also involves the coordinated remodeling of energy substrates including lipids and amino acids. Together, these changes form the metabolic basis for the functional transition of microglia.

The core features of glucose metabolic reprogramming include increased glucose uptake, enhanced glycolytic flux, elevated lactate production, and suppressed mitochondrial oxidative phosphorylation activity. Studies have shown that primary microglia and BV2 cells treated with lipopolysaccharide exhibit high glycolytic activity and low oxidative phosphorylation activity, accompanied by increased lactate production and reduced ATP synthesis (31). In models of Parkinson’s disease (PD), pathological alpha synuclein aggregates are recognized by microglia, triggering a rapid transition from a resting to an activated state. This transition induces significant metabolic reprogramming in the cells, manifested as upregulation of key glycolytic enzymes and mitochondrial dynamics imbalance (32, 33). These changes drive microglia into a sustained proinflammatory metabolic state. This persistent pathological glycolysis not only exacerbates the release of neurotoxic factors but also further activates the NLRP3 inflammasome by promoting succinate accumulation, thereby promoting neuroinflammation and dopaminergic neuron damage in PD (34, 35). Consequently, in relevant diseases, the metabolic state of microglia reflects their functional state and also determines their pathological role in the disease.

The regulatory roles of key metabolic enzymes constitute an important mechanism underlying glycolytic reprogramming. Hexokinase 2 (HK2), a rate limiting enzyme in the glycolytic pathway, is upregulated during microglial activation. This enzyme not only drives microglial activation and neuroinflammation by modulating glycolytic flux but also exerts regulatory functions by influencing mitochondrial activity (36, 37). Studies have shown that specific knockdown of HK2 in microglia inhibits glycolysis and reduces ATP production, thereby limiting microglial proliferation, surveillance function, and injury induced migration (38). Pyruvate kinase M2 (PKM2), another rate limiting enzyme of glycolysis, exhibits increased expression and phosphorylation levels upon proinflammatory stimulation, thereby driving microglial inflammatory responses. PKM2 binds to nuclear factor kappa B as a nuclear transcription factor to increase proinflammatory cytokine transcription (39), and interacts with activating transcription factor 2 to participate in regulating glycolytic metabolism and pyroptosis (40, 41). PKM2 driven glycolysis produces large amounts of lactate, which further amplifies inflammatory responses through histone lactylation. Additionally, 6 phosphofructo 2 kinase/fructose 2,6 bisphosphatase 3 (PFKFB3) is also upregulated in proinflammatory microglia, and its product fructose 2,6 bisphosphate acts as an allosteric activator of phosphofructokinase 1 to promote glycolytic flux (42).

The regulation of glucose metabolism by microglial inflammatory responses involves multiple signaling pathways. Mammalian target of rapamycin (mTOR) is a key factor integrating nutrient and inflammatory signals. Its complex mTORC1 is highly activated in proinflammatory microglia, driving the metabolic shift toward glycolysis by promoting the translation of glycolysis related enzymes and the protein synthesis of hypoxia inducible factor 1 alpha (HIF-1α) (43). HIF-1α is a transcription factor that regulates glycolytic reprogramming. Under inflammatory stimulation, this factor stabilizes and translocates into the nucleus by inhibiting prolyl hydroxylase activity, directly inducing the expression of GLUT1 and GLUT3 as well as glycolytic enzymes such as HK2 and PKM2 (10). Simultaneously, HIF-1α inhibits pyruvate entry into the tricarboxylic acid cycle via pyruvate dehydrogenase kinase. Additionally, AMP activated protein kinase (AMPK) serves as an energy sensor that maintains oxidative phosphorylation under physiological conditions, and when this pathway is inhibited, it promotes glycolytic switching (44). A positive feedback loop exists between metabolites and inflammation. Lactate accumulation resulting from enhanced glycolysis is not merely a byproduct of energy metabolism but also acts as a signaling molecule involved in inflammatory regulation (45). Lactate can increase the transcription of PKM2, lactate dehydrogenase A, and HIF-1α in microglia through histone lactylation, thereby exacerbating inflammatory responses. Succinate, an important intermediate of the tricarboxylic acid cycle, when abnormally accumulated, can stabilize HIF-1α production and act as a ligand to activate the NLRP3 inflammasome, inducing the release of proinflammatory cytokines and aggravating microglial inflammation (10, 46).

## 4 Acupuncture regulates microglial glucose metabolism to suppress neuroinflammation

**Figure 2:**
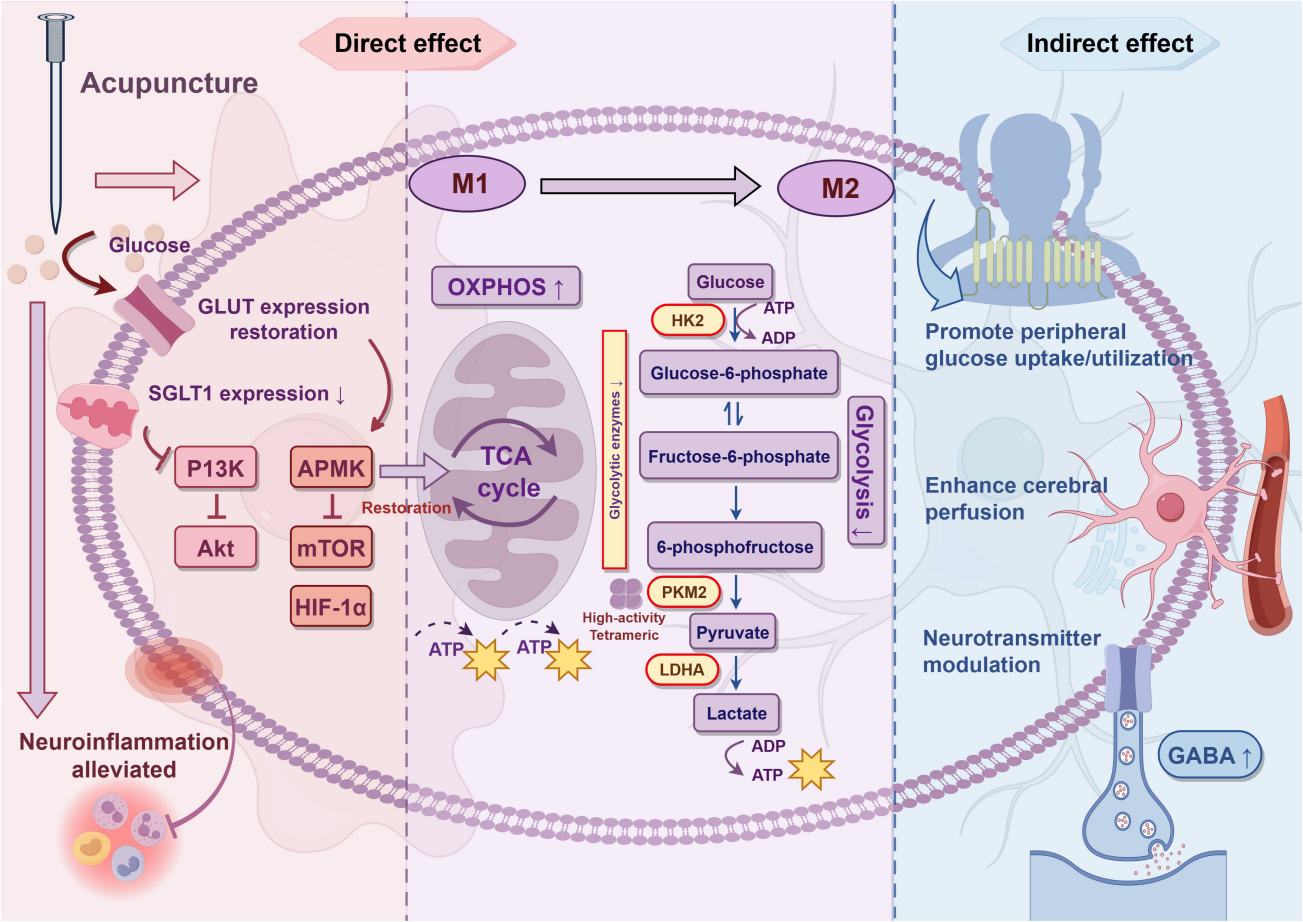
Molecular mechanisms by which acupuncture regulates microglial glycolytic reprogramming to alleviate neuroinflammation. Acupuncture acts through direct and indirect pathways. The direct pathway involves regulating the activity and expression of key glycolytic enzymes, modulating energy sensing signaling pathways, and restoring normal glucose metabolism in microglia. The indirect pathway improves systemic glucose metabolism, optimizes cerebral blood flow and microcirculation, and regulates neurotransmitter balance, thereby remodeling the energy substrate composition and metabolic signals within the brain microenvironment and indirectly guiding the shift in microglial glucose metabolism. The figure was made using www.figdraw.com, and the authorization code is POAYIe572a.

Acupuncture, a classic non pharmacological intervention, has received increasing attention for its anti-inflammatory effects. It may exert neuroprotective effects by regulating microglial glucose metabolic remodeling (**Figure 2**).

### 4.1 Effects on key enzymes of microglial glucose metabolism

GLUT proteins are key mediators of transmembrane glucose transport into microglia. Studies have shown that under neuroinflammatory conditions, microglial GLUT expression is pathologically upregulated, which promotes excessive glucose uptake, thereby driving enhanced glycolytic flux and microglial polarization toward the proinflammatory M1 phenotype (47). *In vitro* experiments confirmed that the GLUT1 specific inhibitor STF31 significantly suppresses microglial glycolytic activity and downregulates proinflammatory cytokine expression (48). In this context, acupuncture can regulate GLUT expression (49). Research has demonstrated that electroacupuncture improves glucose metabolism, modulates GLUT4 expression, and reduces serum levels of IL-6, TNF-α, and C reactive protein (50). Although direct evidence demonstrating changes in GLUT expression in microglia following acupuncture is currently lacking, relevant findings from both peripheral and central studies provide reference for such regulatory effects. Accumulating evidence suggests that acupuncture acts at a systemic level. It indirectly modulates the central microenvironment through the neuro immune metabolic network, thereby influencing the functional state of microglia. Improved glucose metabolism in peripheral tissues reduces circulating levels of pro inflammatory cytokines and abnormal metabolites. These molecules can cross the blood brain barrier and subsequently influence the activity of key glycolytic enzymes in microglia through epigenetic mechanisms such as histone lactylation (51). Meanwhile, the vagus nerve serves as a critical pathway linking peripheral metabolic changes to central neuroimmune responses. Acupuncture effectively activates the vagus nerve, thereby transmitting signals of improved peripheral metabolism to the central nervous system. Through molecules such as the alpha 7 nicotinic acetylcholine receptor, acupuncture further regulates the polarization direction of microglia (52–54). Collectively, the regulation of peripheral glucose metabolism by acupuncture may be converted into indirect modulation of central microglial metabolic reprogramming via this multi pathway integration mechanism.

In a rat model of Alzheimer’s disease (AD), electroacupuncture improved cognitive ability and hippocampal neuronal damage. It increased the cerebral glucose uptake rate and restored the expression of amyloid beta (Aβ) as well as GLUT1, GLUT3, and GLUT4 proteins in hippocampal tissue (55). These effects were mediated through the phosphatidylinositol 3 kinase (PI3K)/protein kinase B (AKT)/glycogen synthase kinase 3 beta (GSK-3β) pathway. Sodium glucose transporters (SGLT) are sodium coupled transporters that absorb glucose and sodium ions into cells. SGLTs regulate glucose metabolism in the central nervous system and protect neuronal function (56). Under electroacupuncture intervention, transient receptor potential channel 1 protein expression is upregulated, Ca^2+^ concentration is increased, and SGLT1 activity is inhibited, thereby promoting glucose metabolism, blocking PI3K/AKT pathway activation, suppressing microglial M1 polarization, and alleviating the progression of PD (57). Although these findings did not directly measure GLUT expression in microglia, the upregulation of GLUT at the hippocampal tissue level and the changes in SGLT may at least partially reflect metabolic adaptations. These adaptations likely occur in microglia and their neighboring cells under the combined influence of central actions and systemic metabolic regulation.

HK2 is the initial rate limiting enzyme of the glycolytic pathway, catalyzing the phosphorylation of glucose to glucose 6 phosphate. HK2 is selectively expressed in microglia, and its deficiency can promote microglial activation, induce neuroinflammation, and exacerbate brain injury (37, 58). In a rat model of multi infarct dementia, the mechanism by which acupuncture improves cognitive impairment is associated with upregulating the activities of HK2, pyruvate kinase, and glucose 6 phosphate dehydrogenase, thereby affecting the energy metabolism system (59). Another study using transcutaneous auricular vagus nerve stimulation, a novel noninvasive acupuncture therapy, reversed microglial activation and alleviated hypothalamic neuroinflammation (60). We hypothesize that acupuncture may exert anti-inflammatory effects by modulating HK2 expression, improving glycolytic activity, and promoting M2 polarization of microglia. PKM2 is a rate-limiting enzyme at the terminal step of the glycolytic pathway, catalyzing the conversion of phosphoenolpyruvate to pyruvate. Unlike other pyruvate kinase isoforms, PKM2 possesses unique conformational plasticity, enabling dynamic switching between low-activity dimers and high-activity tetramers (61). Under physiological conditions, PKM2 predominantly exists as tetramers to efficiently drive glycolysis. However, under pathological stimuli such as hypoxia and inflammation, PKM2 tends to form dimers and translocate to the nucleus, where it acts as a protein kinase and transcriptional coactivator to epigenetically regulate inflammatory gene expression (62). Acupuncture downregulates PKM2 protein and mRNA expression levels, inhibits anaerobic glycolysis in the hippocampus, increases ATP production to meet cellular energy demands, and improves learning and memory function in rats (63). As an end product of glycolysis, lactate induces lactylation modification of PKM2, which suppresses PKM2 dimerization, enhances pyruvate kinase activity, and blocks PKM2 nuclear translocation, thereby driving microglial transition from a glycolysis dependent proinflammatory state to an oxidative phosphorylation dominated anti-inflammatory phenotype (64). However, under chronic neuroinflammatory pathological conditions, this balance is often disrupted. Studies have shown that in AD models, overactive glycolysis leads to lactate accumulation. Lactate subsequently enriches at glycolytic gene promoters via histone H4 lysine 12 lactylation and activates their transcription, thereby further enhancing glycolytic flux. Sustained activation of this positive feedback loop ultimately exacerbates microglial dysfunction, whereas pharmacological inhibition of PKM2 effectively interrupts this process, reduces Aβ burden, and improves cognitive function (45). Therefore, electroacupuncture restores hippocampal neuronal structure in AD mice, reduces the mean optical density of Aβ1-42, downregulates PKM2, lactate dehydrogenase A isoform, HK, and L-lactate content, and improves the colocalization coefficient of lactate dehydrogenase A with glial cells. By acting through the Akt/mTOR/HIF-1α pathway, electroacupuncture ameliorates glycolysis in the hippocampal tissue of AD model mice, thereby restoring cognitive dysfunction (65).

### 4.2 Effects on signaling pathways involved in microglial glucose metabolism

The anti-inflammatory effects of acupuncture on neuroinflammation are not only reflected in the regulation of individual metabolic enzymes but also involve systematic modulation of multiple energy sensing and metabolic signaling pathways within microglia. AMPK, mTOR, and HIF-1α constitute a core signaling axis that governs the balance between glycolysis and oxidative phosphorylation. Under neuroinflammatory conditions, microglia exhibit hyperactivation of HIF-1α and mTOR along with suppressed AMPK activity, an imbalance that drives enhanced glycolytic flux and proinflammatory polarization. Acupuncture intervention can reshape microglial glucose metabolism by activating AMPK while inhibiting excessive activation of mTOR and HIF-1α (50, 65, 66). Furthermore, the PI3K/Akt signaling pathway is also involved in acupuncture mediated regulation of microglial glucose metabolism (55, 57).

### 4.3 Indirect pathways modulating microglial glucose metabolism

Acupuncture regulation of central energy metabolism can also influence the functional state of microglia through multiple indirect pathways. At the systemic metabolic level, acupuncture promotes glucose uptake and utilization in peripheral tissues and optimizes systemic glucose metabolic homeostasis by modulating the activity of the autonomic nervous system and the intrinsic pancreatic neural network, thereby indirectly ensuring substrate supply to the central nervous system (67). At the cerebrovascular level, acupuncture effectively improves regional cerebral blood flow and microcirculatory perfusion, enhancing transmembrane glucose transport and utilization efficiency (68, 69). Furthermore, acupuncture reduces lactate content in ischemic brain tissue and decreases the formation of lactate derived protein lysine lactylation modifications. At the same time, by upregulating the expression of astrocytic MCT1, acupuncture promotes the efflux of intracellular lactate to the extracellular space, thereby maintaining lactate homeostasis in the brain (70). This series of systemic optimizations in metabolism and blood flow reshapes the dynamic balance between cerebral glucose supply and extracellular lactate, indirectly altering the composition of energy substrates available to microglia. Consequently, it reduces compensatory hyperglycolysis triggered by energy deficiency in microglia and effectively suppresses the cascade of neuroinflammation (71). Microglia are capable not only of metabolizing glucose and glutamine but also of uptaking and metabolizing the inhibitory neurotransmitter γ-aminobutyric acid (GABA) (72). In a post-stroke limb spasm model, electroacupuncture intervention increased GABA and IL-10 contents in the ischemic cortex while decreasing glutamate and TNF-α contents, and significantly elevated the protein expression levels of gamma-aminobutyric acid A receptor alpha 1 and glutamate decarboxylase 67. Moreover, the co expression of microglial markers ionized calcium binding adaptor molecule 1, arginase 1, and inducible nitric oxide synthase was restored to normal levels, indicating that acupuncture reshapes the GABA/glutamate balance and thereby modulates the metabolic microenvironment surrounding microglia at the neurotransmitter level, ultimately alleviating neuroinflammation (73). Meanwhile, acupuncture also modulates the noradrenergic signaling pathway (74). Neurotransmitter signals induced by acupuncture act on corresponding receptors on the microglial surface, subsequently activating intracellular energy sensing pathways and initiating downstream mitochondrial related programs. This promotes a shift in microglial energy acquisition from a compensatory glycolysis dominated mode to an efficient high capacity mode centered on mitochondrial oxidative phosphorylation (71).

## 5 Summary and outlook

### 5.1 Summary of the study

This article systematically reviews the pathophysiological mechanisms of microglial glycolytic reprogramming in neuroinflammation and explores the potential scientific basis of acupuncture in regulating this process. The functional state of microglia is closely linked to their metabolic profile. Under physiological conditions, microglia maintain efficient energy metabolism through oxidative phosphorylation, whereas under pathological stimulation of neuroinflammation, they undergo metabolic reprogramming toward a glycolysis dominated proinflammatory mode. This shift is not merely a concomitant phenomenon of microglial activation but rather an important upstream mechanism driving the initiation and persistence of neuroinflammation. Recent studies have achieved preliminary progress in understanding how acupuncture regulates microglial glucose metabolism to inhibit neuroinflammation. Acupuncture can systemically reshape microglial glucose metabolism by modulating GLUT expression, intervening in the activity and expression of key glycolytic enzymes such as HK2 and PKM2, and regulating multiple energy sensing signaling pathways including AMPK/mTOR/HIF-1α and PI3K/AKT. In addition, acupuncture improves the composition of energy substrates and metabolic signals within the brain microenvironment through various indirect pathways, including enhancing systemic glucose metabolism, optimizing cerebral blood flow and microcirculation, and regulating neurotransmitter balance, thereby indirectly guiding the transition of microglial glucose metabolic states.

### 5.2 Limitation analysis

However, current research still faces many bottlenecks that urgently need to be addressed. The regulatory effects of acupuncture on central metabolism have a preliminary research basis. Nevertheless, direct evidence of microglial metabolic remodeling following acupuncture intervention remains relatively weak. Most existing studies have focused primarily on the expression levels of key glycolytic enzymes. There is still a lack of *in situ* and real time monitoring techniques for dynamic changes in microglial glucose metabolism. This methodological limitation makes it difficult to accurately determine whether acupuncture exerts its effects by modulating glycolytic flux, promoting the restoration of oxidative phosphorylation, or altering substrate utilization preferences.

To objectively present the current evidence base, this review adopts a grading system for critical evaluation of the relevant literature. Direct evidence is defined as observed changes in target metabolic molecules at the brain tissue level, with the experimental design at least involving microglia. Indirect evidence refers to detected changes in metabolic pathways or glucose transporters in whole brain tissue lysates, but without cell type specific validation. Speculative hypotheses are those that report metabolic improvements only at the peripheral or systemic level. These hypotheses then infer effects on microglia through known associative mechanisms such as the vagus nerve, blood brain barrier, or systemic metabolic optimization. Such inferences lack direct support at the central or cellular level. **Table 1** systematically categorizes the included studies according to this grading system. In summary, few studies currently meet the criteria for direct evidence. These studies have observed regulatory effects of acupuncture on key glycolytic enzymes such as GLUT, PKM2 and HK2, as well as related pathways, at the brain tissue level. However, this direct evidence remains relatively direct only at the *in vivo* tissue level. It is not sufficient to establish a causal relationship between acupuncture and microglial glucose metabolism reprogramming. Indirect evidence constitutes a larger number of studies. These findings indicate that acupuncture regulates central glucose transporter expression and the activity of the AMPK/mTOR/HIF-1α signaling pathway. However, because brain tissue samples contain multiple cell types including neurons and astrocytes, these observations only provide indirect validation for microglia specific effects. Speculative hypotheses account for the largest proportion of studies. They suggest that acupuncture improves systemic glucose metabolism, peripheral inflammatory cytokines, blood brain barrier integrity, and neurotransmitter balance. These findings provide important background for understanding the multi level effects of acupuncture. Nevertheless, these systemic changes cannot be directly equated to acupuncture mediated reprogramming of microglial glucose metabolism. This represents a major limitation of the present review and warrants further refinement, supplementation, and validation in future studies.

**Table 1:** Overview of studies on acupuncture regulation of microglial glucose metabolism.

Disease model	Acupoint selection	Acupuncture modality (stimulation parameters)	Outcome measures	Evidence category	Reference
T2DM rat	Sanyinjiao (SP6), Zusanli (ST36)	EA (2 mA, 2/100 Hz, dense-sparse wave	Increased GLUT4 expression, reduced inflammatory cytokine levels, and regulated insulin signaling pathways	SH	(50)
AD rat	Baihui (GV20), Shenting (GV24)	EA (1 mA, 2/20 Hz, dense-sparse wave)	Increased GLUT1, GLUT3, and GLUT4 expression, regulated the PI3K/AKT/GSK-3β pathway, and improved glucose metabolism level in the brain	IE	(55)
MPTP mouse	The chorea trembling control area of the head	EA (0.2 mA, 2Hz)	Inhibited SGLT1 effect, facilitated glucose metabolic regulation, blocked activation of PI3K/AKT pathway, and suppressed microglial M1 polarization	DE	(57)
MID rat	Danzhong (CV17), Zhongwan (CV12), Qihai (CV6), Xuehai (SP10), Zusanli (ST36)	MA	Upregulated HK2, PK, and glucose-6-phosphate dehydrogenase activity, thereby improving energy metabolism and cognitive function	SH	(59)
CUMS-exposed rat	The distribution area of the auricular branch of the vagus nerve	taVNS (2 ms square pulses, 2 mA, 2/15 Hz)	Modulated the hypothalamic α7nAChR/JAK2/STAT3/NF-κB signaling pathway, and suppressed microglial M1 polarization	SH	(60)
MCAO/R rat	Shenting (GV24), Baihui (GV20)	EA (≤6 V, 1/20 Hz, dense-sparse wave)	Inhibited the HIF-1α/PDK1 pathway, reduced the expression of HK2, PFK1, and PKM2 in the hippocampal tissue, increased ATP production, and inhibited anaerobic glycolysis	IE	(63)
AD mouse	Baihui (GV20), Zusanli (ST36), Shenshu (BL23)	EA (1 mA, 2 Hz, continuous wave)	Inhibited the hippocampal Akt/mTOR/HIF-1α signaling pathway, reduced the expression of PKM2, LDHA, HK, and L-lactic acid, and decreased the co-localization coefficient of LDHA with glial cells	DE	(65)
AD mouse	Baihui (GV20)	EA (1/20 Hz, disperse waves)	Activated the AMPK/AKT signaling pathway, increased the expression of GLUT1 and GLUT3, and improved the glucose metabolism in the hippocampal tissue	IE	(66)
T2DM rat	Tianshu (ST25)	EA (2 mA, 2/15 Hz)	Regulated the hypoglycemic function of the intrinsic pancreatic nervous system	SH	(67)
MID rat	Danzhong (CV17), Zhongwan (CV12), Qihai (CV6), Xuehai (SP10), Zusanli (ST36)	MA	Increased cerebral blood flow and improved mitochondrial function and the endogenous oxidative stress system in the brain	SH	(68)
MCAO/R rat	Neiguan (PC6)	EA (1 mA, 4/20 Hz, sparse-density wave)	Improved cerebral blood flow in the CA1 region of the hippocampal tissue and increased the expression of GLUT1	IE	(69)
MCAO/R rat	Neiguan (PC6), Quchi (LI11)	EA (1 mA, 2/15 Hz, dense-sparse wave)	Increased MCT1 expression in astrocytes, promoted lactate transport, and improved neural function	IE	(70)
PSS rat	Yanglingquan (GB34), Quchi (LI11)	EA (100 Hz, dense wave)	Regulated M1-to-M2 microglial polarization, reduced inflammatory cytokine levels, and reduced the Glu/GABA ratio	SH	(73)

SH, speculative hypotheses; IE, indirect evidence; DE, direct evidence; EA, electroacupuncture; MA, manual acupuncture; T2DM, type 2 diabetes; GLUT, glucose transporters; AD, Alzheimer’s disease; PI3K, phosphatidylinositol 3 kinase; AKT, protein kinase B; GSK-3β, glycogen synthase kinase 3 beta; MPTP, 1-methyl-4-phenyl-1,2,3,6-tetrahydropyridine; SGLT, sodium glucose transporter; MID, multi-infarct dementia; HK, hexokinase; PK, pyruvate kinase; CUMS, chronic unpredicted mild stress; taVNS, transcutaneous auricular vagus nerve stimulation; α7nAchR, α7 nicotinic acetylcholine receptor; JAK2, janus kinase 2; STAT3, signal transducer and activator of transcription 3; NF-κB, nuclear factor kappa-B; MCAO/R, middle cerebral artery occlusion/reperfusion; HIF-1α, hypoxia-induced factor-1α; PDK1, pyruvate dehydrogenase kinase 1; PFK1, phosphofructokinase 1; mTOR, mammalian target of rapamycin; LDHA, lactate dehydrogenase A; APMK, Adenosine 5**’**-monophosphate (AMP)-activated protein kinase; MCT1, monocarboxylate transporter 1; PSS, post-stroke spasticity; Glu, Glutamic acid; GABA, gamma-aminobutyric acid.

Emerging evidence also highlights the interconnectedness of systemic metabolic regulation and central neuroinflammation, supporting a broader, multi-organ perspective on disease modification (75). Glucose metabolism represents only one aspect of acupuncture mediated regulation of microglial energy metabolism, as lipid metabolism and amino acid metabolism also play important roles in neuroinflammation. Lipid metabolic disturbances in microglia can lead to accumulation of neurotoxic lipids and activation of proinflammatory signaling pathways, whereas amino acids such as glutamine serve as critical alternative substrates for microglia under glucose limited conditions. These metabolic pathways form a complex regulatory network with glucose metabolism, collectively constituting the metabolic foundation for the functional transition of microglia. Current studies predominantly focus on the single dimension of glucose metabolism, with insufficient attention given to the cross talk and coordinated remodeling among multiple metabolic pathways in microglia. Whether acupuncture concurrently regulates lipid and amino acid metabolism, and how these metabolic changes synergize with glycolytic remodeling to exert anti-inflammatory effects, remain to be thoroughly investigated.

Notably, the characteristics of microglial metabolic reprogramming differ across pathological stages. Whether acupuncture acts through identical or similar metabolic regulatory mechanisms under different conditions currently lacks systematic comparative studies. Furthermore, the regulation of microglial glucose metabolism is dynamic (10), and different glycolytic patterns may exist during the acute versus chronic phases of neuroinflammation (31). Whether acupuncture acts through identical or similar metabolic regulatory mechanisms under different pathological contexts also remains unreported. After reviewing the available literature, we found that acupuncture upregulates HK activity in the multi-infarct dementia model (59). In contrast, under chronic neuroinflammatory conditions such as the middle cerebral artery occlusion/reperfusion (MCAO/R) and AD models, acupuncture reduces HK overexpression. It thereby drives microglial anti inflammatory polarization by inhibiting glycolysis (63, 65). These findings suggest that the regulatory effect of acupuncture on HK2 may be dependent on both the pathological stage and the baseline metabolic state. In pathological states characterized by low basal glucose metabolism, acupuncture may restore overall energy supply by upregulating HK2. This supports the basal immune surveillance function of microglia. Conversely, in chronic inflammatory states with excessive glycolysis activation, acupuncture may downregulate HK2 or suppress its overactivity. This intervention can then interrupt the positive feedback loop between glycolysis and inflammation. Therefore, current studies may not yet fully elucidate the precise regulatory mechanisms by which acupuncture acts on key glycolytic enzymes. Further in depth research is needed to clarify this issue.

### 5.3 Future prospects

Synthesizing the available evidence, several acupoints are commonly used across various models such as AD, MCAO/R, and post stroke spasticity. Baihui (GV20) is widely selected. Shenting (GV24) is often combined with GV20 to enhance central effects. In addition, trunk acupoints such as Zusanli (ST36), Sanyinjiao (SP6), and Zhongwan (CV12) are also applied. This combination suggests an indirect influence on the central microenvironment through regulation of systemic metabolism. Regarding stimulation methods, most studies employ electroacupuncture, particularly with dense-sparse wave stimulation, while a few disease models use manual acupuncture. These electroacupuncture protocols activate peripheral and central metabolic regulatory networks. These findings may help optimize acupoint selection and stimulation parameters for acupuncture treatment of neuroinflammatory diseases.

In addition, previous studies have shown that certain active ingredients of traditional Chinese medicine can regulate microglial glucose metabolism by targeting metabolic enzymes. Whether acupuncture can exert synergistic effects when combined with these agents is an area worthy of further exploration. In terms of combination therapy, acupuncture shows synergistic potential with existing drugs. Electroacupuncture combined with the active component of traditional Chinese medicine, trigonelline, can synergistically inhibit pyroptosis and autophagy in cerebral ischemia reperfusion injury (76). Manual acupuncture combined with donepezil is superior to either intervention alone in improving cognitive function and cerebral glucose metabolism in the AD model (77). These findings suggest that the systemic multi target effects of acupuncture complement the blocking effects of small molecule inhibitors. Regarding the interaction between acupuncture and metabolic targeted agents such as HK2 inhibitors or PKM2 modulators, there may be a combined effect of synergistic enhancement and functional complementarity. To date, no study has evaluated the combined effect of acupuncture with the above metabolic targeted agents in neuroinflammatory models. This represents a direction for future mechanistic exploration. Furthermore, clinical studies should be conducted to validate the exact mechanisms of microglial glucose metabolism under acupuncture intervention, thereby achieving an effective translation from basic research to clinical application.

## 6 Conclusion

Overall, although this review incorporates a substantial amount of indirect evidence and speculative hypotheses, current research still provides an interpretable framework for the mediating mechanism by which acupuncture targets microglial glucose metabolism reprogramming to regulate neuroinflammation. Future studies should further investigate the regulatory mechanisms of acupuncture on key glycolytic enzymes and signaling pathways in microglia to address the current limitation of insufficient direct evidence. Simultaneously, integrating dynamic monitoring of both glycolytic remodeling and inflammatory responses will help elucidate the causal and temporal relationship between metabolic changes and immune regulation underlying the anti-inflammatory effects of acupuncture. Multi-omics technologies should be employed to systematically dissect the acupuncture mediated metabolic network in microglia, revealing its multi-target and multi pathway metabolic remodeling characteristics across glucose, lipid, and amino acid metabolism. As research in immunometabolism advances, targeting microglial metabolic reprogramming by acupuncture holds promise for opening new avenues and strategies for the prevention and treatment of diseases.
